# Clinical Profile and Management of Pleural Effusion at Tertiary Hospital of Nepal: An Observational Study

**DOI:** 10.31729/jnma.9155

**Published:** 2025-07-31

**Authors:** Amit Kumar Singh, Robin Man Karmacharya, Satish Vaidya, Shrijana Singh, Ishu Shrestha, Prabha Bhandari, Gakul Bhatta, Sonu Basnet, Subash Neupane

**Affiliations:** 1 Department of General Surgery, Nepal National Hospital, Kalanki, Kathmandu; 2 Department of General Surgery, Dhulikhel Hospital, Kathmandu University Hospital, Dhulikhel, Nepal; 3 Department of General Surgery, James Paget Hospital NHS Trust, UK; 4 Newham University Hospital, NHS Trust, UK; 5 Institute of Crisis Management Studies, Kathmandu, Nepal.

**Keywords:** *chest-tube*, *parapneumonic effusion*, *pleural effusion*, *thoracocentesis*, *tubercular effusion.*

## Abstract

**Introduction:**

Pleural effusion is the accumulation of excessive fluid in the pleural cavity. Despite the high incidence of pleural effusion in Nepal, there is limited data regarding the clinical profile and its management. The study aims to find out the clinical profile and management of pleural effusion in a tertiary care hospital.

**Methods:**

This is a descriptive cross-sectional study done over 24 months in patients with pleural effusion admitted to a tertiary care hospital in Nepal. Ethical clearance was taken from the Institutional Review Committee (No: 134/20). Census sampling was done. All patients who were diagnosed to have pleural effusion were included in the study. The clinical findings, radiological imaging reports, management record and relevant data of each case were retrieved from the medical records. Data were analysed using Statistical Package for the Social Sciences 25.

**Results:**

Among 273 cases ofpleural effusion, 186 (68.1%) were male. The mean age of the patients was 48.83±22.87 and 97 (35.53%) were above 60 years. The pleural effusion on right side was 137 (50.93%). Among all cases, 241 (88.3%) were managed conservatively; 72 (29.88%) with antitubercular therapy and 169 (70.12%) without antitubercular therapy, while surgical management was required in 32 (11.7%) cases.

**Conclusions:**

The most common cause of pleural effusion was tubercular in origin; majority of cases can be managed conservatively, and only particular cases require surgical interventions like chest tube placement or thoracotomy.

## INTRODUCTION

Pleural effusion is the accumulation of excess fluid in the pleural cavity and is a major global cause of morbidity.^1-3^ It results from an imbalance between fluid formation and removal, often indicating underlying pulmonary or systemic diseases like heart or renal failure. Effusions are classified as transudative or exudative.^4^ Annually, about 1.5 million cases are reported in the U.S.^5^ In Nepal and India, tuberculosis is the leading cause,whereas in Western countries, 90% are due to CCF, pneumonia, malignancy, or viral infections.^6-9^ Effusions develop in 20-40% of bacterial pneumonias and up to 87% of heart failure patients.^10,11^ Management aims at symptom relief and treating the cause.^2,3^ Transudates often resolve with conservative therapy, while exudates may require drainage, Anti-tubercular Therapy (ATT), or surgery.^10^ Pleural fluid cultures are often negative (90-98%).^1^2 Malignancies cause 18-20% of cases, mainly from lung, breast, ovary, or lymphoma.^12,13^ Despite its clinical burden, Nepal lacks comprehensive data on effusion profiles, necessitating this study.

## METHODS

This descriptive retrospective cross-sectional study was conducted over a period of twenty-four months, from January 2019 to February 2021, at Dhulikhel Hospital, a tertiary care teaching hospital affiliated with Kathmandu University. The study aimed to assess the clinical profile and management patterns of pleural effusion in patients admitted during the study period. Prior to data collection, ethical clearance was obtained from the Institutional Review Board of the hospital (Reference number: 134/20).

All patients who presented to the hospital and were diagnosed with pleural effusion were included in the study, irrespective of age and gender. Inclusion criteria encompassed patients with newly diagnosed pleural effusion based on clinical, radiological, and laboratory evidence. However, patients who were intubated, hemodynamically unstable, or had previously undergone pleural tapping or drainage before admission were excluded from the study to maintain diagnostic consistency and data integrity.

The diagnosis of pleural effusion was made using a combination of clinical symptoms (such as dyspnea, chest pain, and cough), physical examination findings (including decreased breath sounds, dullness to percussion, and reduced chest expansion), radiological imaging (chest X-ray, ultrasound, or CT scan), and supportive laboratory investigations, including analysis of pleural fluid obtained through thoracocentesis.

A total of 273 patients who met the inclusion criteria during the study period were included through census sampling.

All relevant clinical information was obtained retrospectively by reviewing patient records from the hospital’s medical record section and centralized hospital information system (HIS). Each patient was evaluated using available clinical history, physical examination findings, laboratory test results, radiological reports, and treatment records. Diagnostic investigations included routine blood tests, sputum analysis for AFB, and pleural fluid analysis, which consisted of biochemical (protein, LDH, glucose), cytological, and microbiological parameters. These were performed during the course of clinical management and recorded for analysis. Information on the type of management (conservative or surgical), use of anti-tubercular therapy (ATT), and surgical interventions such as chest tube insertion or thoracotomy was also extracted. The extracted data were first entered into Microsoft Excel and subsequently exported to Statistical Package for the Social Sciences (SPSS), version 25.0 for Windows (IBM Corp., Armonk, NY, USA) for statistical analysis.

Descriptive statistics were used to summarize the demographic and clinical characteristics of the study population. Categorical variables (nominal variables) such as gender, laterality of effusion, etiology, and management type were expressed as frequency and percentage. Continuous variables (scalar variables) such as age were presented as mean, standard deviation (SD), and range.

All findings were interpreted in the context of existing literature, and no inferential statistics were applied due to the descriptive nature of the study.

## RESULTS

A total of 273 patients with a diagnosis of pleural effusion were included in this study over the 24-month period. Among them, 186 patients (68.1%) were male, while 87 (31.9%) were female, resulting in a male-to-female ratio of approximately 2.1:1. The mean age of presentation was 48.83 ± 22.87 years, with the age range spanning from 8 to 94 years. Patients aged above 60 years of age accounted for 97 (35.53%) cases, followed by the 41-60 years group with 76 (27.83%) patients (Table 1).

**Table 1:** Age group of the patients with pleural effusion (n = 273)

Variables	n(%)
Gender	
Male	186(68.10)
Female	87(31.90)
Age	
< 20 years	38(13.91)
20–40 years	62(22.83)
41-60 years	76(27.83)
> 60 years	97(35.53)

**Table 2:** Site involvement of pleural effusion (n=273).

Site	n(%)
Left	76(27.80)
Right	140(51.03)
Bilateral	57(20.91)

**Table 3:** Clinical profile of pleural effusion (n = 273)

Diagnosis	n(%)
Tubercular effusion	83 (30.40)
Parapneumonic effusion	72 (26.37)
Malignant effusion	37 (13.55)
Congestive heart failure	22 (8.05)
Renal disease	12 (4.39)
Liver disease	11 (4.02)
Others	36 (13.18)
History of Tuberculosis	
Yes	9 (3.30)
No	264 (96.70)
Family History of Tuberculosis	
Yes	2 (0.70)
No	271 (99.30)

**Figure 1:**
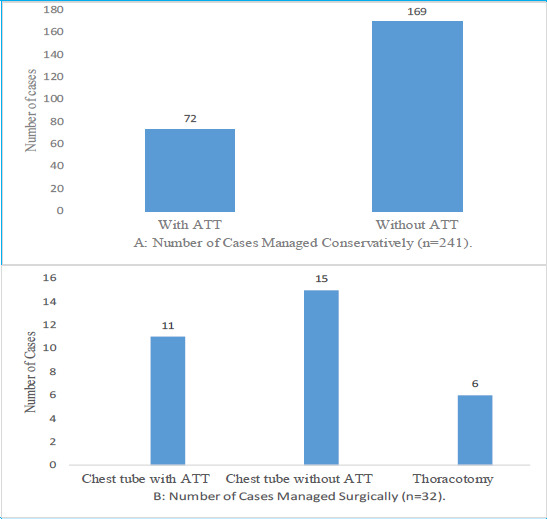
Management of pleural effusion included both conservative and surgical approaches (n=273).

Among all cases of pleural effusion, 140 (51.3%) patients had pleural effusion confined to the right hemithorax, whereas 76 (27.80%) patients had effusion on the left side, and 57 (20.91%) patients presented with bilateral pleural effusion (Table 2). Among all cases of pleural effusion, tubercular effusion accounted for 83 (30.40%) cases and parapneumonic effusion accounted for 72 (26.37%) cases. There were 9 (3.30%) patients with history of tuberculosis and 2 (0.70%) patients with family history of tuberculosis (Table 3).

Out of all cases, 241 (88.27%) were managed conservatively, and surgical intervention was performed in 32 cases (11.73%). Among patients managed conservatively, 72 (29.88%) received antitubercular therapy (ATT) and 169 (70.12%) did not. Among surgically managed patients, 15 (46.87%) underwent chest tube insertion without ATT, 11 (34.37%) underwent chest tube insertion with ATT, and 6 (18.75%) underwent thoracotomy (Figure 1).

## DISCUSSION

This study presents a detailed overview of the clinical presentation and management of pleural effusion in a tertiary care hospital in Nepal, offering important insights into disease patterns in a resource-limited setting. One of the most notable findings is the predominance of tubercular pleural effusion, which accounted for nearly one-third of all cases (30.4%). This aligns with existing evidence from Nepal and other high tuberculosis (TB) burden countries, where TB continues to be a leading cause of pleural effusions.^5,7^ The high proportion of TB-related effusions reflects not only the ongoing public health challenge posed by tuberculosis in Nepal, but also the critical need for robust diagnostic and treatment frameworks for managing extrapulmonary TB manifestations.

In addition to tuberculosis, parapneumonic (26.4%) and malignant effusions (13.6%) also emerged as significant causes, aligning with the etiological spectrum described in studies conducted in both South Asia and Globally.^6,7,12^ Parapneumonic effusions are typically associated with bacterial pneumonias and often require prompt recognition to prevent progression to empyema. Malignant effusions, although less frequent, represent a clinically challenging group due to their recurrent nature and association with poor prognosis. Compared to data from Western countries where congestive heart failure, malignancy, and pulmonary embolism are leading cause of pleural effusion; the pattern observed in this study highlights the distinct epidemiological landscape of South Asia, where infectious causes like TB remain dominant.^8^ These regional differences emphasize the importance of context-specific clinical guidelines and diagnostic algorithms when approaching pleural effusion in different parts of the world.

One of the most notable aspects of this study is the overwhelming reliance on conservative treatment approaches, with more than 88% of patients managed without the need for surgery. This trend reflects a growing recognition that many pleural effusions particularly those that are uncomplicated can be effectively treated with simple interventions such as thoracocentesis combined with appropriate medical therapy. These findings align with previous studies, which have emphasized that invasive procedures are often unnecessary when timely diagnosis and treatment are provided.^9,10^

The high success rate of non-surgical management in our cohort reinforces the idea that conservative care not only works but also helps to reduce patient burden, hospital stay, and procedural risks an especially important consideration in settings with limited access to surgical services.^11^ When surgical intervention was required, chest tube drainage emerged as the most commonly used method, consistent with global standards for managing more complex effusions, such as those associated with infection or tuberculosis.^12-14^

Additionally, our data revealed a slight right-sided predominance in pleural effusion cases (51.03%). While the exact cause of this asymmetry remains uncertain, it mirrors findings from other studies in the region. Possible explanations may include anatomical differences between the two pleural cavities or varying patterns in disease involvement, but further investigation is warranted to better understand this observation. ^5^

In our study, a clear male predominance was observed, with 68.1% of patients being male, and the majority of participants falling within the age group of 60 years and above. This finding is in line with other regional studies, including one by Bhavsar and Pujara, which also reported a higher incidence of pleural effusion among older male patients.^2^ Several factors could contribute to this trend, including gender-based differences in occupational exposures, health-seeking behavior, and underlying risk factors such as smoking or chronic respiratory conditions.

Interestingly, despite a large proportion of elderly participants, the mean age in our study was relatively young at 48.8 years, which suggests that pleural effusion is not limited to the geriatric population alone. This is particularly relevant in the context of Nepal, where tuberculosis and parapneumonic effusions; two of the leading causes identified in this study tend to affect individuals in their most productive years. The implications are significant, not only from a clinical and epidemiological perspective but also for public health planning and socioeconomic impact, as the disease may lead to prolonged absence from work, reduced productivity, and added financial burden on families and the healthcare system.

Despite the meaningful insights, this study has several limitations. The retrospective design and single-centre scope limit the generalizability of the findings. The reliance on clinical and radiological diagnosis, with limited microbiological confirmation due to the high rate of negative pleural fluid cultures as reported by Ranjit et al.may affect diagnostic precision. Moreover, convenience sampling could introduce selection bias. ^15^

Despite its limitations, this study provides valuable insights with practical relevance for clinicians working in similar settings. In a country like Nepal, where healthcare resources are limited and access to advanced diagnostic tools may not be universally available, adopting a conservative-first treatment strategy for pleural effusion proves to be both a sensible and sustainable approach. Most patients in this study responded well to conservative management, suggesting that with careful clinical assessment and timely initiation of appropriate therapy particularly Anti-Tubercular Therapy when indicated many cases can be effectively managed without the need for invasive procedures.

These findings also serve as a reminder of the critical need to strengthen tuberculosis diagnostic and treatment pathways, especially since TB continues to be the most common underlying cause of pleural effusion in this context. Early identification and prompt initiation of therapy can prevent complications and reduce the burden on tertiary healthcare facilities.

Looking ahead, there is a strong case for conducting prospective, multi-center studies across various regions of Nepal and other low- and middle-income countries.

## CONCLUSION

This study concludes that tuberculosis remains the most common cause of pleural effusion in our setting, followed by parapneumonic and malignant effusions. A majority of patients can be effectively managed with conservative treatment, including Anti-Tubercular Therapy where indicated, with surgical interventions reserved for selected cases. These findings emphasize the importance of timely diagnosis and a context- appropriate treatment approach. Strengthening clinical decisionmaking and enhancing access to basic diagnostics can further improve patient outcomes in similar resource-limited settings.

## Data Availability

The data are available from the corresponding author upon reasonable request by the editorial team.
